# Sex‐ and APOE Genotype–Dependent Pain Susceptibility and Alzheimer's Risk Mediated by the Lipid Metabolism Enzyme LPCAT2

**DOI:** 10.1111/acel.70234

**Published:** 2025-09-17

**Authors:** Rai‐Hua Lai, Ren‐Hua Chung, Wan‐Yu Pai, Yi‐Chung Chen, Ka‐Hei Lam, Cheng‐Nong Lai, Jyh‐Lyh Juang

**Affiliations:** ^1^ National Center for Geriatrics and Welfare Research National Health Research Institutes Miaoli Taiwan; ^2^ Institute of Molecular and Genomic Medicine National Health Research Institutes Miaoli Taiwan; ^3^ Institute of Population Health Science National Health Research Institutes Miaoli Taiwan; ^4^ Institute of Biochemistry and Molecular Biology Chinal Medical University Taichung Taiwan

**Keywords:** Alzheimer's disease (AD), APOE genotype, LPCAT2, mild cognitive impairment (MCI), pain susceptibility, sex‐specific differences

## Abstract

Neuropathological changes that precede or accompany early cognitive decline in Alzheimer's disease (AD) may also impact pain processing; however, the molecular connection between these domains remains unclear. In this study, we investigated whether a shared causal factor underlies both increased pain susceptibility and AD progression. Analysis of two ethnically distinct cohorts revealed a significant association between pain susceptibility and cognitive decline from cognitively normal (CN) status to mild cognitive impairment (MCI), particularly in non‐*APOE4* (non‐E4) males, an unexpected finding given that *APOE4* females exhibited the highest overall pain susceptibility across sex and genotype groups. To explore potential drivers of this *APOE* genotype‐ and sex‐specific association, blood transcriptomic analysis identified *LPCAT2* expression as correlating with both heightened pain susceptibility and progression from MCI to AD, most notably in non‐E4 males. This relationship was further supported by elevated LPCAT2 protein levels in the hippocampus of postmortem non‐E4 male AD patients. Strengthening this link, our genetic association analysis across four cohorts identified several functional *LPCAT2* variants that not only influenced its expression but were also associated with altered pain susceptibility, increased AD risk, and accelerated progression from MCI to AD. To move beyond correlation and assess causality, Mendelian randomization analysis supported a causal role for LPCAT2 in both pain susceptibility and MCI‐to‐AD progression. Collectively, these findings identify LPCAT2 as a key molecular link between altered pain processing and AD progression, highlighting its potential as both a therapeutic target for genotype‐ and sex‐specific subpopulations and a prognostic biomarker for MCI‐to‐AD conversion.

## Graphical abstract

We aimed to uncover the molecular mechanisms linking age‐related pain susceptibility with cognitive decline in Alzheimer's disease (AD). By integrating transcriptomic, human AD brain, genomic, and Mendelian randomization analyses, we identified the lipid‐modifying enzyme LPCAT2 as a sex‐ and *APOE* genotype–dependent causal mediator of both heightened pain vulnerability and AD progression.

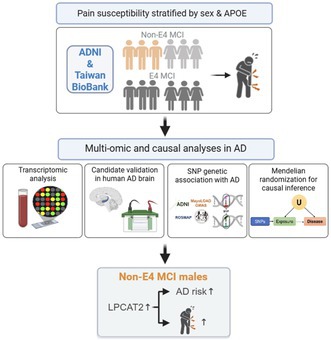

## 1 Introduction

Clinically, progressive cognitive decline and cerebral atrophy are the hallmark pathological features of AD (Ballard et al. 2011). However, emerging evidence indicates that noncognitive symptoms, such as altered pain susceptibility, mood disturbances, and sleep disruptions, often precede the onset of overt dementia. This observation suggests that the cellular mechanisms driving these early manifestations might also modulate AD initiation and progression (Brzecka et al. 2018; Eikelboom et al. 2021; Zhao et al. 2023). Notably, altered pain susceptibility has recently attracted significant interest as a potential early biomarker for AD, offering a unique window into the underlying neuropathological mechanisms.

Changes in pain susceptibility during the early stages of AD or MCI may arise from neuropathological alterations in pain‐processing regions, such as the insula and anterior cingulate cortex, as well as broader disruptions in cortical connectivity (Apkarian et al. 2009; Yang and Chang 2019). Notably, neuropathic pain affects approximately 9.6% of the general population but may impact up to 50% of individuals with AD (Bornier et al. 2023; Zhao et al. 2023). Pain susceptibility not only diminishes quality of life but may also accelerate cognitive decline and exacerbate neurobehavioral dysfunction (Michaelides and Zis 2019; Woo 2010). Managing chronic pain in AD is particularly challenging due to impaired communication, cognitive deficits, and the complexity of pain processing in a neurodegenerating brain (Achterberg et al. 2020).

Emerging mechanistic insights suggest that lipid metabolism serves as a crucial link between AD and chronic pain (Jin et al. 2020; Yin 2023). Dysregulated lipid signaling has been implicated in both neuroinflammatory responses in AD and nociceptive hypersensitivity in chronic pain models (Domenichiello and Ramsden 2019). For instance, aberrant phospholipase A2 activity and subsequent elevations in prostaglandins are among the earliest biochemical changes observed in AD brains, contributing to neuroinflammation and neuronal damage (Bazan et al. 2002). Similarly, lysophosphatidylcholine (LPC) has been strongly associated with neuropathic pain (Ren et al. 2022). Elevated LPC levels promote immune activation and demyelination, leading to persistent pain hypersensitivity in both animal models and human patients (Freeman et al. 2017; Plemel et al. 2018; Ren et al. 2022).

Various genetic, behavioral, environmental, and sex‐related factors have been linked to both pain susceptibility and AD. Among these, *APOE* genotype and sex are especially critical, as they shape distinct biological profiles that influence both pain susceptibility and AD progression. Specifically, emerging evidence suggests that carriers of *APOE4*, a major genetic risk factor for AD, exhibit altered pain intensity and sensitivity compared to noncarriers (Romano et al. 2021), potentially due to structural and functional changes in pain‐processing regions, such as the anterior cingulate cortex and insula (Monroe et al. 2012; Romano et al. 2021; Tansley et al. 2022). Likewise, sex differences are also well documented in both AD risk and pain perception (Aggarwal and Mielke 2023; Fan et al. 2020). Clinically, females report higher pain susceptibility and prevalence than males, driven by hormonal fluctuations, immune responses, and pain‐related gene expression (Casale et al. 2021). Postmenopausal women, in particular, face a greater risk of AD, exhibit more rapid cognitive decline, and show distinct patterns of brain atrophy, underscoring the need for sex‐specific approaches. These findings highlight *APOE*‐ and sex‐dependent differences in pain perception and AD risk, emphasizing the importance of considering these variables for developing targeted interventions. However, the precise mechanisms linking *APOE* genotype and sex to pain susceptibility and their impact on AD pathogenesis remain largely unknown.

This study examines the causal links between pain susceptibility and AD progression, with a particular focus on lysophosphatidylcholine acyltransferase 2 (LPCAT2), a key enzyme involved in lipid metabolism and inflammation. By elucidating these connections, our findings may pave the way for the development of novel diagnostic and prognostic biomarkers as well as therapeutic strategies to delay or mitigate AD progression.

## 2 Materials and Methods

### 2.1 Data Sources: ADNI, Taiwan Biobank, ROSMAP, and MayoLOADGWAS Repositories

This study utilized data from the Alzheimer's Disease Neuroimaging Initiative (ADNI) database (https://adni.loni.usc.edu/), including blood microarray profiles, single nucleotide polymorphism (SNP) genotyping data, clinical diagnostic information, and questionnaire‐based assessments. Our study included a total of 1153 participants diagnosed with MCI or classified as CN. Detailed demographic and baseline characteristics of the subjects are presented in Table S1. Data concerning musculoskeletal pain were obtained from the “ADSXLIST.csv” and “BLSCHECK_18Sept2022.csv” files, whereas blood microarray data were obtained from the “microarray.csv” file. Information including the time from progression of MCI to AD, dementia diagnosis date, cognitive function, and *APOE* genotype was extracted from the “ADNIMERGE.csv” file. Genetic data were retrieved from the “ADNI Omni2.5M microarray SNP data” set. Participants who received an AD diagnosis within 3 months of their initial MCI classification were excluded from the analysis to avoid potential diagnostic misclassification. All ADNI data used in this study were accessed and downloaded in November 2022.

For the Taiwan BioBank (https://www.twbiobank.org.tw/), we utilized genetic data, pain perception measures (specifically, reports of joint pain as an indicator of musculoskeletal discomfort), Mini‐Mental State Examination (MMSE) scores, and baseline demographic and health‐related information. These data were obtained from the “TWB1 and TWB2 imputed combined genetic data (GRCh38)” dataset, as well as the “release_list_measure.csv” and “release_list_survey.csv” files. Detailed demographic and baseline characteristics of the participants are provided in Table S2. All data were accessed in October 2023.

For the Religious Orders Study/Memory and Aging Project (ROSMAP) dataset (https://doi.org/10.7303/syn3219045) and The Mayo Clinic LOAD genome‐wide association and brain gene expression (MayoLOADGWAS) study (https://doi.org/10.7303/syn2910256), we utilized genetic data, as well as information on age and AD diagnosis. Additionally, we incorporated data from the “ROSMAP_clinical.csv” file and “ROSMAP arrayGenotype” PLINK binary files from the (ROSMAP) dataset, as well as the “mayoloadgwas_snpgenotypes_covariates.csv” and “mayoloadgwas_snpgenotypes” PLINK binary files from the (MayoLOADGWAS) dataset. All datasets were obtained and accessed in February 2025.

Access to each repository adhered to its data use agreement and custodian policies. All data were deidentified prior to access, and no reidentification was attempted. Data collection and secondary use complied with applicable data‐protection regulations, including the EU General Data Protection Regulation (GDPR) where relevant, Taiwan's Personal Data Protection Act and Human Subjects Research Act, and US human subjects regulations (e.g., the Common Rule). Original study cohorts obtained informed consent for broad scientific use, and our access was conducted under controlled‐access terms. All data were stored on encrypted, access‐controlled servers, accessible only to authorized study personnel, and all analyses were performed on deidentified datasets. Repository data can be obtained through their standard application procedures; the summary results from this study are provided in the Supporting Information and are available from the corresponding author upon reasonable request, subject to the relevant data‐use agreements.

For the murine cortical and hippocampal transcriptomics analysis, data from the cortical and hippocampal regions of male WT and male APP/PS1 mice were analyzed, adapted from MENDELEY DATA (https://doi.org/10.17632/z9264694b4.2) (Papazoglou et al. 2023). Expression levels of *LPCAT2* were assessed using two probe IDs: A_51_P169061 and A_55_P2809820.

### 2.2 Analysis of Pain Susceptibility Across Cognitive States, Stratified by *APOE4* Genotype and Sex

To investigate the association between pain susceptibility and cognitive status during the progression from CN to MCI, data from the ADNI cohort were analyzed. Participants were classified based on cognitive status (CN or MCI). Given the established influence of *APOE4* genotype and sex on pain perception, further stratification was performed by *APOE4* carrier status (carriers vs. noncarriers) and sex (male vs. female) to examine potential subgroup differences. Pain experience was assessed using self‐reported measures from the ADNI dataset. The proportion of individuals reporting musculoskeletal pain was calculated for each subgroup, and group differences were evaluated using a two‐tailed Fisher's exact test.

To assess the relationship between pain experience and cognitive function, data from older adults in the Taiwan BioBank were analyzed. Cognitive function was measured using Mini‐Mental State Examination (MMSE) scores (ranging from 18 to 30, with scores of 18–23 indicating possible MCI and 24–30 indicating normal cognition) (Tombaugh and McIntyre 1992), and pain experience was determined based on questionnaire responses. A linear regression analysis was conducted to examine the association between pain and MMSE scores, adjusting for age. Analyses were further stratified by *APOE4* carrier status (carriers vs. noncarriers) and sex (male vs. female). Regression coefficients (*B*), *p* values, and 95% confidence intervals (CIs) were reported to evaluate the strength and significance of the association.

### 2.3 Transcriptomics Identification of Genes Associated With Pain and AD Onset in MCI Individuals

To identify differentially expressed genes associated with both pain susceptibility and AD onset in non‐E4 males, microarray gene expression analysis was performed on whole blood samples from CN and MCI individuals in the ADNI cohort. Genes exhibiting significant differential expression (log_2_ fold change ≥ 0.4 or ≤ −0.4 and *p* < 0.05) in MCI were further intersected with gene sets linked to pain perception and AD progression, identifying candidate genes relevant to both conditions.

To explore the relationship between gene expression and pain perception in MCI, whole blood transcript levels of *LPCAT2* and additional candidate genes were analyzed. A total of 370 MCI participants were stratified based on self‐reported pain experiences. For each gene, expression levels were classified as “high” or “low” relative to the mean expression level across the ADNI cohort. Group differences in gene expression were assessed using an unpaired two‐tailed *t*‐test, with statistical significance set at *p* < 0.05. All statistical analyses were performed using GraphPad Prism 10.0.

To assess the relationship between *LPCAT2* expression levels and the rate of AD progression, longitudinal cognitive data from 439 individuals with MCI were analyzed using data obtained from the ADNI cohorts. Participants were stratified into high‐ and low‐expression groups for *LPCAT2* or other candidate genes based on their median transcript levels. The progression rate to AD was evaluated using standardized clinical cognitive assessments over time. Kaplan–Meier survival analysis was employed to compare the time to AD conversion between the two expression groups, with statistical significance determined using a two‐tailed log‐rank test (*p* < 0.05).

### 2.4 Human Brain Tissue and Ethics Statement

Human postmortem brain tissues were sourced from the NICHD Brain and Tissue Bank for Developmental Disorders, currently integrated into the NIH NeuroBioBank. A total of 37 brain tissues were utilized, as previously described (Lai et al. 2021). *APOE* genotyping was performed using western blot analysis with an anti‐ApoE4 antibody (Cell Signaling Technology, Cat# 8941) and confirmed by DNA sequencing. The *APOE* genotype was determined by DNA sequencing. The primers used for amplification and sequencing were as follows: *APOE*_sequencing_F (forward): 5′‐GAC CAT GAA GGA GTT GAA GGC CTA C‐3′ and *APOE*_sequencing_R (reverse): 5′‐CTC GCG GGC CCC GGC CTG GTA‐3′. All studies and protocols were approved by the Research Ethics Committee at National Health Research Institutes (approved protocol no. EC1001103).

### 2.5 Mice

Double transgenic APP/PS1 AD mice (Cat# 037565‐JAX, RRID: MMRRC_037565‐JAX) were obtained from The Jackson Laboratory and bred with wild‐type B6C3F1/Bltw mice. Mice were housed under controlled conditions with a 12‐h light/dark cycle and a constant ambient temperature of 25°C to ensure optimal living conditions. Brain tissues were collected from 7‐month‐old AD mice and their wild‐type (WT) counterparts. Hippocampal tissue was dissected and subjected to quantitative real‐time reverse transcription‐polymerase chain reaction (qRT‐PCR) to assess *LPCAT2* expression levels. All animal procedures were approved by the Institutional Animal Care and Use Committee (IACUC) of the National Health Research Institutes (NHRI) under protocol numbers NHRI‐IACUC‐101057‐A and NHRI‐IACUC‐103136‐A.

### 2.6 Quantitative Real‐Time Reverse Transcription‐Polymerase Chain Reaction Analysis

Total RNA was extracted from brain tissues or cultured cells using the FavorPrep Blood/Culture Cell Total RNA Isolation Kit (GE Healthcare Life Sciences) according to the manufacturer's protocol. Reverse transcription was performed using the High‐Capacity cDNA Reverse Transcription Kit (ABI Applied Biosystems). qRT‐PCR was conducted using Fast SYBR Green Master Mix (ABI Applied Biosystems), and gene expression levels were analyzed using standard curve quantification. The following primers were used for qRT‐PCR analysis: Mouse_LPCAT2_Forward: 5′‐CTT CGC TGG TAT CAC GGA ATG AG‐3′; Mouse_LPCAT2_Reverse: 5′‐CCA TTC TCC ACC TGA TGT CGC T‐3′; Mouse_GAPDH_Forward: 5′‐CAT CAC TGC CAC CCA GAA GAC TG‐3′; Mouse_GAPDH_Reverse: 5′‐ATG CCA GTG AGC TTC CCG TTC AG‐3′.

### 2.7 Association Analysis of *LPCAT2* SNPs With Pain Perception and AD Risk in ADNI and ROSMAP

To quantify pain experience and AD risk, each individual's pain score in the ADNI dataset was represented by pain frequency (high vs. low; *n* = 812) or categorized based on clinical diagnosis (MCI/AD vs. CN; *n* = 737). Participants were stratified by *APOE* genotype and sex. A threshold of 0.465, corresponding to the average pain frequency across individuals with available genetic data, was used to define high versus low pain experience. Two separate SNP analyses were performed using the PLINK 1.90 whole‐genome association toolset. The first analysis focused on pain perception among individuals with MCI/AD, who were grouped based on high or low pain frequency to identify *LPCAT2*‐related SNPs associated with pain experience. The second analysis examined AD risk, with CN individuals as controls and MCI/AD individuals as cases. ROSMAP and MayoLOADGWAS data were used to assess the association between *LPCAT2* SNPs and AD‐related phenotypes.

### 2.8 Genetic Association Analysis of *LPCAT2* SNPs With Pain Susceptibility and Cognitive Decline in Taiwan BioBank

For the analysis with the Taiwan BioBank dataset, *LPCAT2* SNPs significantly associated with *LPCAT2* gene expression levels were selected to investigate their relationship with pain experience and cognitive function (MMSE score). Initially, we identified all SNPs in the *LPCAT2* gene with significant associations with *LPCAT2* gene expression based on eQTL results obtained from the Genotype‐Tissue Expression (GTEx) Portal (https://gtexportal.org/). We then selected the SNPs in *LPCAT2* present in the Taiwan BioBank for further association analysis. Participants were categorized into two groups based on their questionnaire responses: those with pain experience and those without (*n* = 1828). The association of pain frequencies with different *LPCAT2* SNPs was calculated using PLINK, and a chi‐square test was employed to determine the significance of differences between the pain experience and no pain experience groups. For cognitive function (*n* = 741), SNPs were classified into three groups based on their genotypes: minor allele homozygous, heterozygous, and major allele homozygous. ANOVA analysis was used to determine differences among these groups.

### 2.9 Mendelian Randomization

To assess the potential causal role of LPCAT2 in Alzheimer's disease (AD) pathophysiology, we conducted Mendelian randomization (MR) analyses using four SNPs located within the LPCAT2 gene (rs9922534, rs1362399, rs1502003, and rs1502004), each located in distinct linkage disequilibrium (LD) regions. These SNPs were selected based on their significant associations with LPCAT2 mRNA expression levels in blood, which served as the exposure variable. Two outcomes were examined: pain perception, assessed by pain frequency scores in the ADNI dataset, and AD progression, defined as the time (in months) from initial diagnosis of MCI to conversion to AD. Genotype data and matched expression levels were available for all included participants. MR analyses were performed using multiple complementary approaches, including inverse‐variance weighted (IVW), weighted median, simple median, penalized IVW, and robust MR estimation methods, implemented via the MendelianRandomization R packages (Burgess et al. 2013). These methods were used to account for potential pleiotropy, heterogeneity, and outlier effects. For each model, we reported the estimated causal effect, standard error, 95% confidence interval, and statistical significance.

### 2.10 Statistical Analysis

Statistical analyses were conducted using GraphPad Prism 10.0, DataTab (https://datatab.net/), and PLINK 1.90, depending on the data type and analytic purpose. Baseline characteristics, including sample size (*n*), mean or median values, and statistical comparisons, are described in the respective tables and figure legends. For continuous variables, comparisons between two groups were conducted using two‐tailed unpaired *t*‐tests. One‐way analysis of variance (ANOVA) was used for comparisons among more than two groups (e.g., MMSE scores across different genotypes). For categorical variables, chi‐square tests were applied when group sizes were adequate, and two‐tailed Fisher's exact tests were used for small sample sizes (e.g., subgroup comparisons of pain experience across *APOE4* status and sex). SNP association analyses were performed using PLINK 1.90. Analyses included comparisons of allele distributions across pain experience groups and cognitive performance strata. Kaplan–Meier survival analysis was used to evaluate the time to AD conversion, and statistical differences between groups were tested using two‐tailed log‐rank tests. In box‐and‐whisker plots, the box edges indicate the lower and upper quartiles (Q1 and Q3), with the interquartile range (IQR) representing the central 50% of the data. The line within the box denotes the median value.

## 3 Results

### 3.1 *APOE* Genotype and Sex Influence Pain Susceptibility in MCI

**FIGURE 1:**
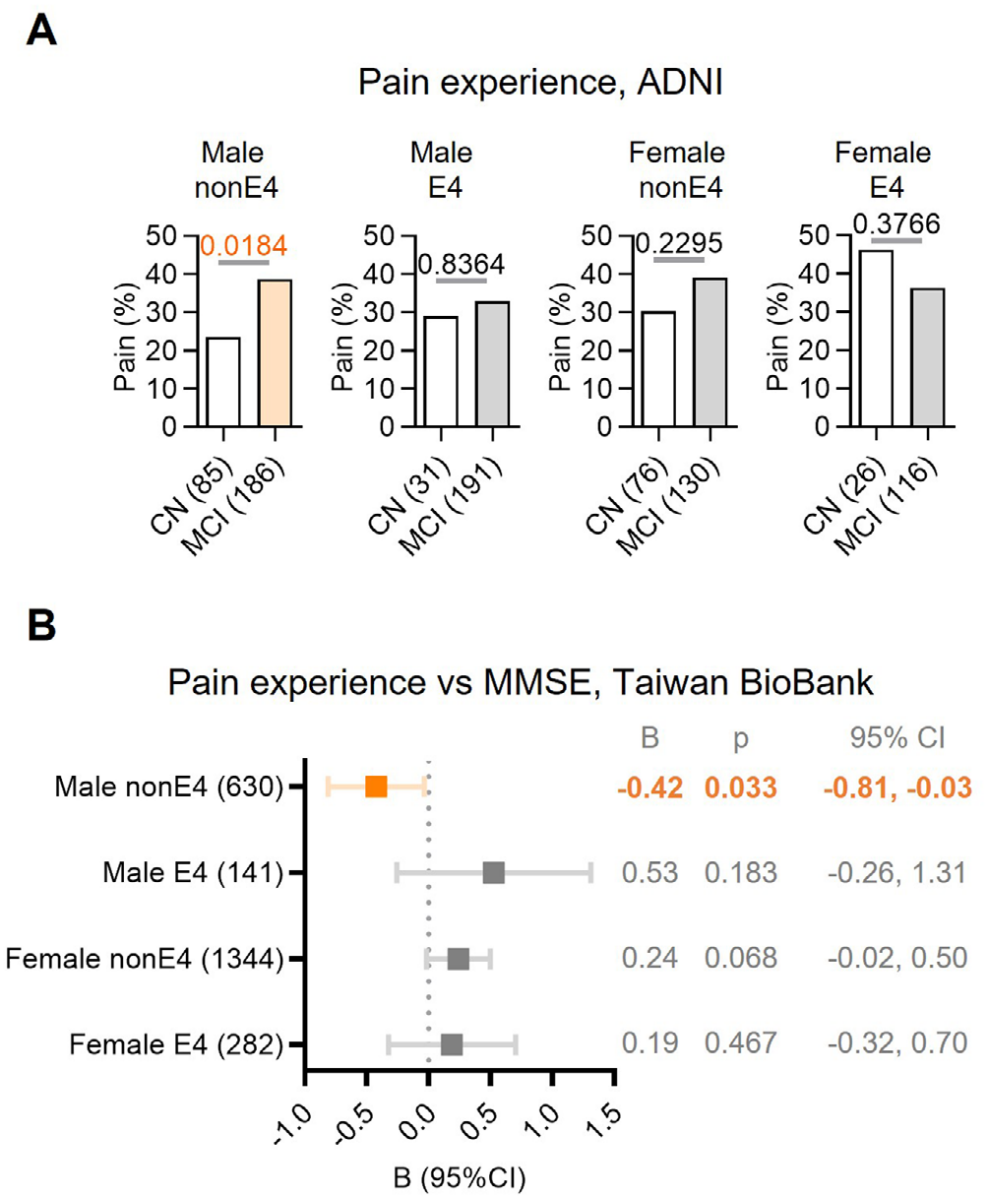
Association of pain susceptibility with *APOE4* and sex interaction during cognitive decline. (A) Musculoskeletal pain prevalence across cognitive states, stratified by *APOE* genotype and sex. The prevalence of musculoskeletal pain during the CN and MCI stages was analyzed in male and female subjects with or without the *APOE4* genotype in the ADNI cohort. Statistical significance was determined using a two‐tailed chi‐square test, with *p* < 0.05 considered significant (non‐E4: non‐*APOE4* carriers; E4: *APOE4* carriers). Numbers in parentheses after group labels indicate sample sizes. (B) Association between pain susceptibility and MMSE scores in elderly subjects from the Taiwan BioBank. Linear Regression analysis was performed to assess the relationship between MMSE scores and musculoskeletal pain in elderly subjects from the Taiwan BioBank. The model was adjusted for age to control for potential confounding effects. *B*, Regression coefficient; *p*, *p* value; 95% CI, 95% confidence interval. Numbers in parentheses after group labels indicate sample sizes.

Altered pain susceptibility has been observed in early AD patients before cognitive symptoms emerge; however, it remains unclear whether this phenomenon is influenced by or dependent on *APOE* genotype and sex. By analyzing the ADNI database, the results revealed that although *APOE4* CN females exhibited the highest pain perception across groups differentiated by *APOE4* status and sex, their pain susceptibility was not significantly altered in MCI. In contrast, we identified a significant association between pain susceptibility change specifically in non‐E4 males (Fisher's exact test, *p* = 0.0184). In contrast, we did not find a statistically significant association between pain and progression to MCI in non‐E4 females and *APOE4* carriers (Figure 1A). This result suggests that pain susceptibility is significantly amplified during the cognitive decline only in non‐E4 males.

We further validated this finding in an independent ethnic cohort from the Taiwan BioBank. Cognitive function, assessed by MMSE scores as a potential predictor of MCI, was analyzed for its impact on pain perception across subgroups stratified by sex and *APOE4* genotype (Figure 1B and Table S2). After adjusting for age, a statistically significant association between cognitive function and pain perception was observed in non‐E4 males (*B* = −0.42, 95% CI = −0.81 to −0.03, *p* = 0.033), while no significant associations were found in the other subgroups. These results align with our observations from the ADNI database, further highlighting that the relationship between pain susceptibility and cognitive decline is particularly pronounced in non‐E4 males across different ethnic populations.

### 3.2 Transcriptomics Links *LPCAT2* to Pain and AD Risk in non‐E4 MCI Males

**FIGURE 2:**
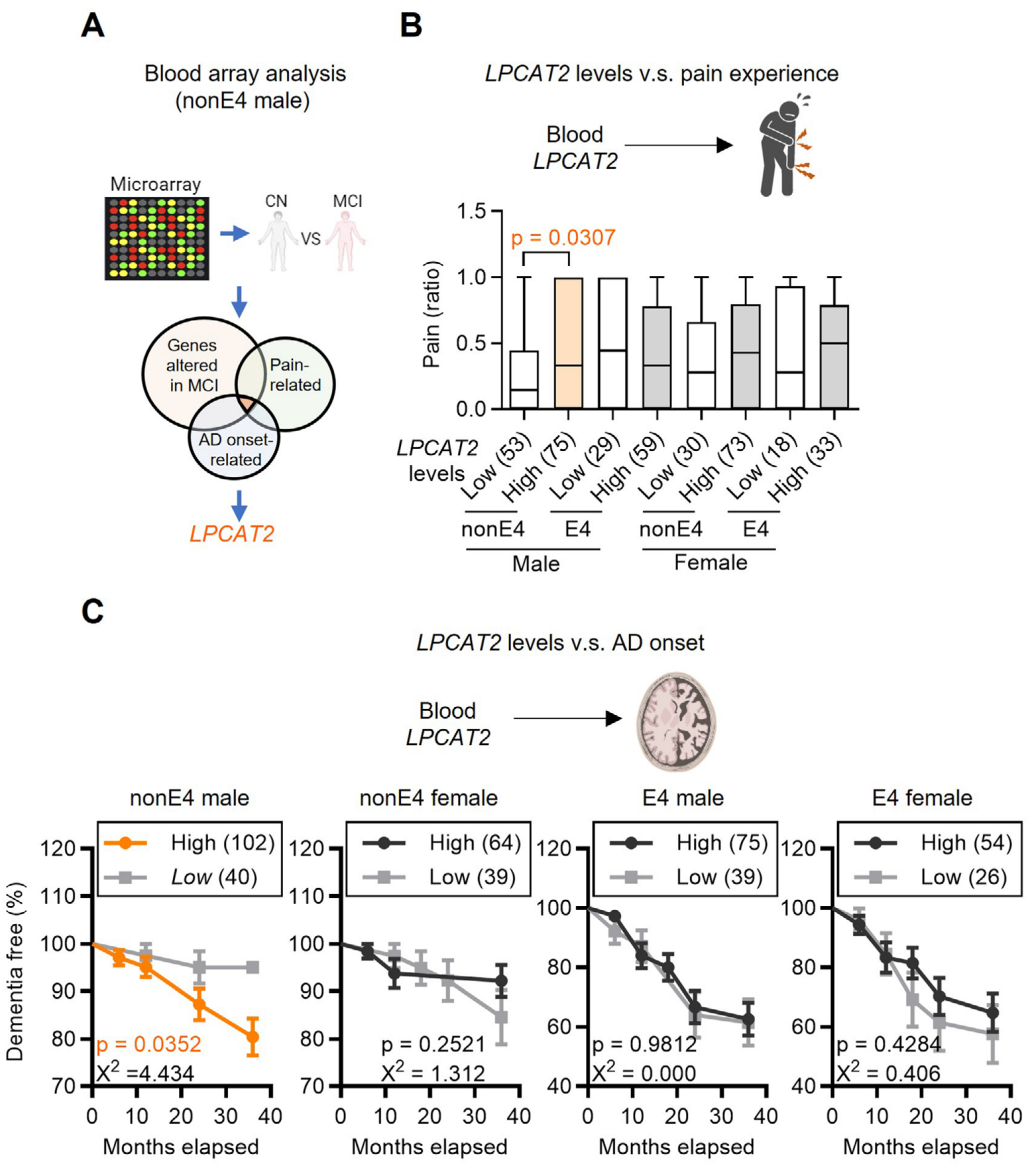
L*PCAT2* levels are associated with pain susceptibility and AD onset in non‐*E4* males. (A) Identification of *LPCAT2* as a pain‐ and AD‐related gene in MCI. Microarray analysis comparing CN and MCI subjects identified differentially expressed genes associated with both pain susceptibility and AD onset. *LPCAT2* was highlighted as a gene significantly linked to both pain experience and early AD pathology. Genes exhibiting significant differential expression were defined by a log_2_ fold change ≥ 0.4 or ≤ −0.4 and *p* < 0.05. (B) Association between *LPCAT2* levels and pain susceptibility. The relationship between *LPCAT2* expression levels and self‐reported musculoskeletal pain experience was analyzed. *LPCAT2* levels were categorized as low (≤ 6.131) and high (> 6.131). Statistical significance was determined using a two‐tailed chi‐square test, with *p* < 0.05 considered significant (non‐E4: non‐APOE4 carriers; E4: *APOE4* carriers). Numbers in parentheses after group labels indicate sample sizes. (C) Association between *LPCAT2* levels and AD progression speed. The impact of *LPCAT2* expression on the speed of AD onset was analyzed using a two‐tailed log‐rank test, with *p* < 0.05 considered significant. Numbers in parentheses after group labels indicate sample sizes.

Previous studies have identified several cellular factors linked to pain susceptibility, particularly those involved in neurotransmitter signaling, inflammation, and central nervous system (CNS) pain processing (Yennurajalingam et al. 2021). However, the relationship between these pain‐related genes and AD remains largely unclear. To explore the connection between pain and MCI onset, we focused on identifying key cellular factors that influence pain susceptibility during the transition from CN to MCI in non‐E4 males. We analyzed peripheral blood microarray gene expression data from the ADNI cohort (Figure 2A) to identify genes associated with both pain susceptibility and subsequent progression to AD at the MCI stage, based on the premise that circulating immune cells may reflect systemic molecular responses to CNS pathologies related to pain and neurodegeneration (Jain et al. 2024; McCaffrey 2006). Our analysis identified several gene transcripts, including *LPCAT2*, *CPA3*, *CASP8*, *GATA2*, and *HDC*, that were significantly associated with the CN‐to‐MCI transition in non‐E4 males (Table S3).

To pinpoint the key gene among the five candidates, we examined whether their expression levels were associated with both pain perception and AD risk. Among them, *LPCAT2*, *CPA3*, and *GATA2* showed associations with pain susceptibility; however, only *LPCAT2* expression was also linked to AD onset (Figures S1 and S2). Notably, *LPCAT2* expression was significantly correlated with AD onset specifically in non‐E4 males with MCI, regardless of *APOE4* status or sex (Figure 2B,C). In contrast, no significant associations were observed in non‐E4 females or *APOE4*‐positive males and females during the transition from CN to MCI and AD. These findings suggest that *LPCAT2* may underlie the observed link between pain susceptibility and AD onset in non‐E4 males.

### 3.3 Postmortem Brain Analysis Reveals Elevated LPCAT2 Protein Levels in non‐E4 Male AD Brains

**FIGURE 3:**
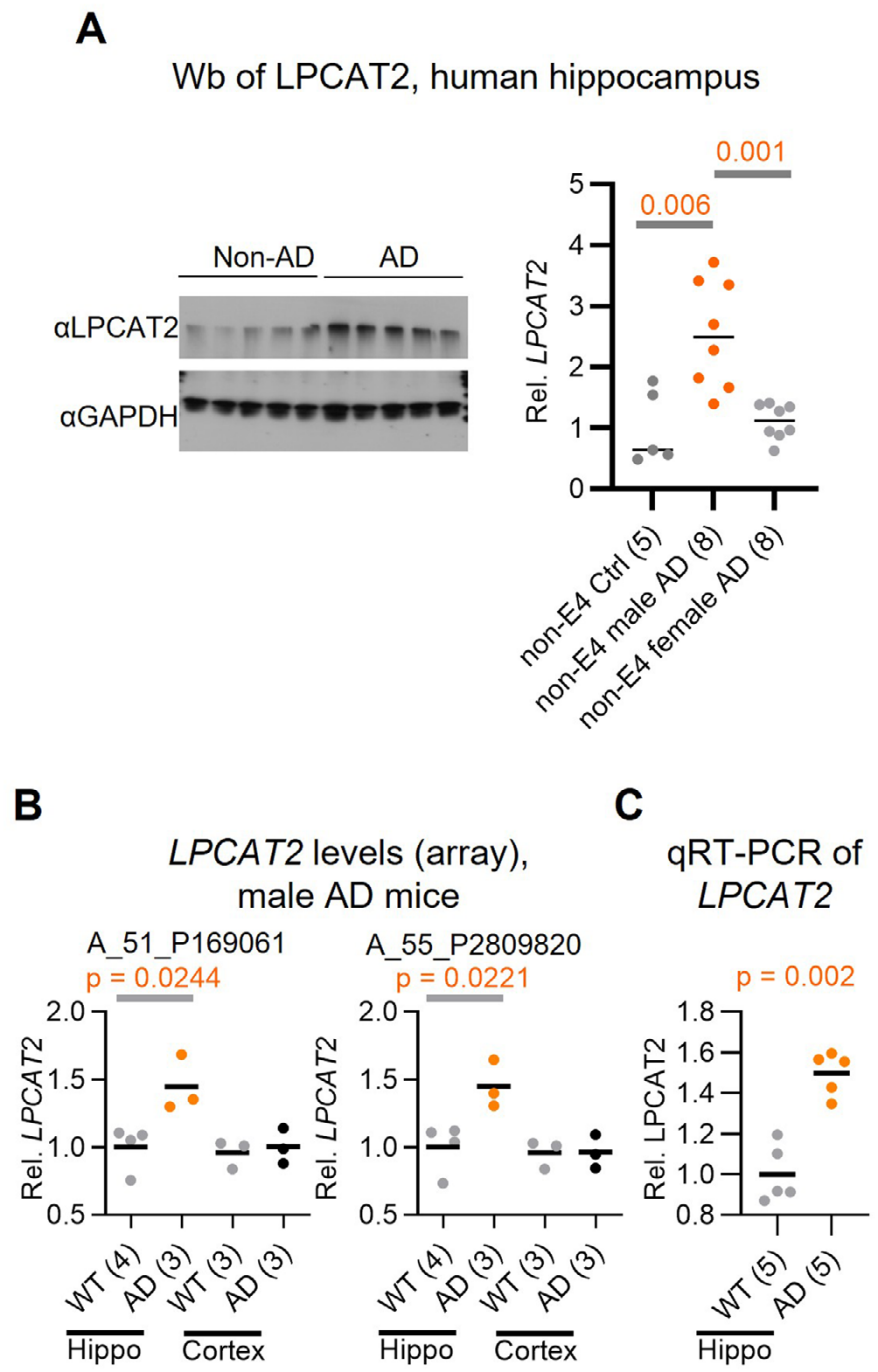
Increased LPCAT2 levels in the hippocampus of non‐E4 AD males and APP/PS1 AD mice. (A) Western blot analysis of hippocampal tissues from human postmortem. LPCAT2 protein levels were detected using specific antibodies, with GAPDH as a loading control. Quantification indicates significantly higher LPCAT2 expression in AD cases (*p* = 0.0031). (B) *LPCAT2* transcript levels in the cortical (Cortex) and hippocampal (Hippo) regions of male wild‐type (WT) and APP/PS1 AD mice. Microarray data were analyzed from MENDELEY DATA (https://doi.org/10.17632/z9264694b4.2) using two probe IDs: A_51_P169061 and A_55_P2809820. *LPCAT2* expression was significantly elevated in AD mice (*p* = 0.0244 in A_51_P169061x; *p* = 0.0221 in Hippo). Statistical significance was determined using a two‐tailed *t*‐test with *p* < 0.05 considered significant. (C) *LPCAT2* expression levels in male APP/PS1 AD mice. Hippocampal *LPCAT2* mRNA levels were measured by qRT‐PCR. Numbers in parentheses after group labels indicate sample sizes.

Building on the identification of LPCAT2's roles in pain susceptibility and AD onset in clinical data, we aimed to validate these findings using human brain tissue samples. We assessed LPCAT2 protein levels in postmortem human hippocampal samples. Consistent with our observations in peripheral blood, LPCAT2 protein levels were significantly elevated, particularly in the brains of non‐E4 male AD subjects compared to non‐AD controls. In contrast, no significant changes were observed in females or *APOE4* male subjects (Figure 3A and Figure S3). These findings highlight a potential role for brain LPCAT2 in the pathogenesis of AD within this subgroup. Consistent with this, LPCAT2 levels were significantly elevated in the hippocampal tissue of APP/PS1 AD mice compared to wild‐type (WT) controls (Figure 3B,C). These results indicate that, in addition to its elevation in peripheral blood, LPCAT2 is also markedly upregulated in the hippocampus during AD progression, particularly in non‐E4 patients.

### 3.4 Genetic Polymorphism Analyses Reveal *LPCAT2* Genetic Variants Link to Pain Susceptibility and AD Risk

**FIGURE 4:**
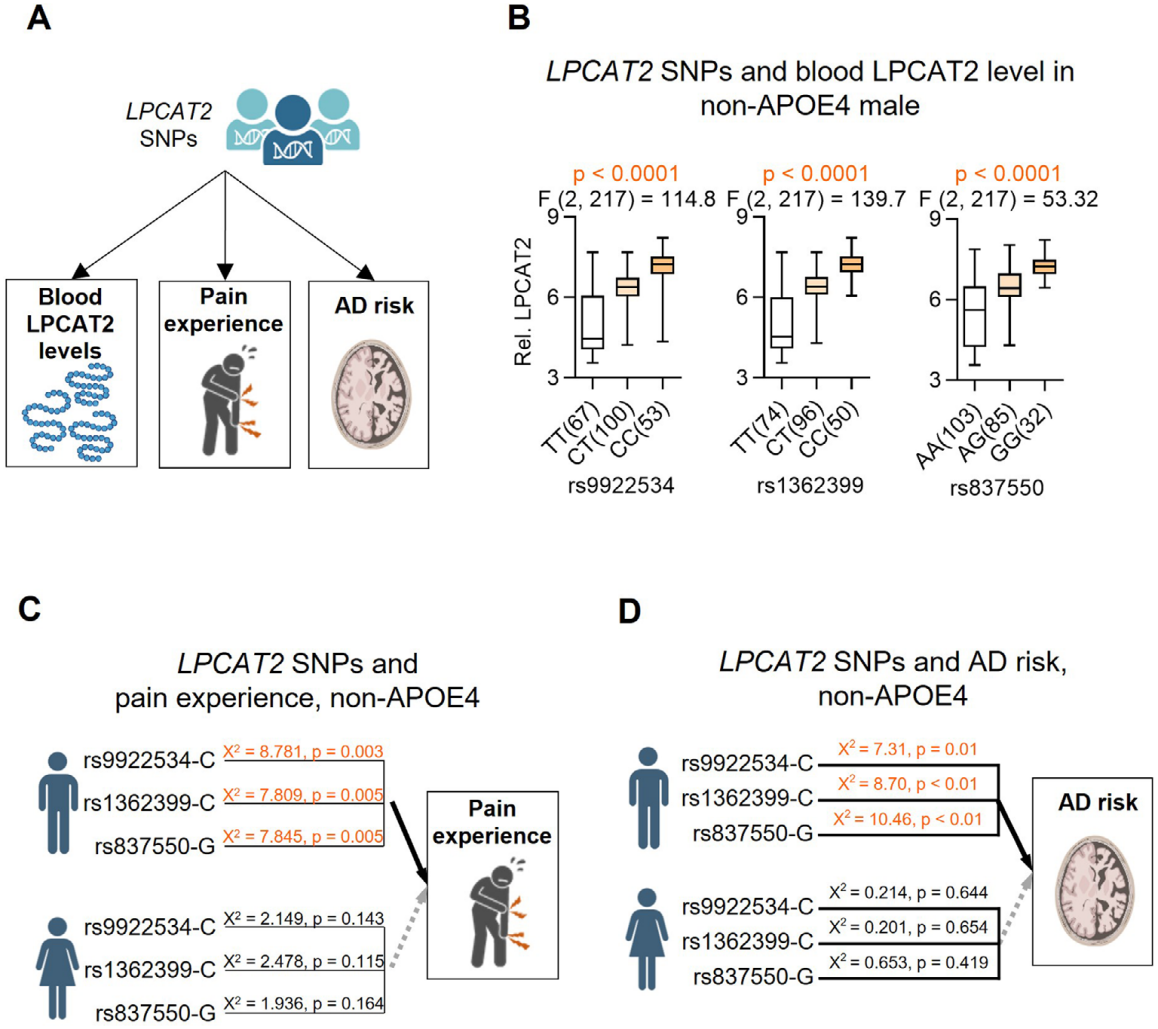
Genetic association of *LPCAT2* SNPs with blood *LPCAT2* levels, pain experience, and AD risk in ADNI. (A) Study overview of the genetic association analysis. A schematic illustration of the study design assessing the relationship between *LPCAT2* single nucleotide polymorphisms (SNPs), blood *LPCAT2* levels, pain experience, and Alzheimer's disease (AD) risk. (B) Association of *LPCAT2* SNPs with blood *LPCAT2* levels. Genetic associations between the top three *LPCAT2* SNPs (rs9922534, rs1362399, and rs837550) and blood *LPCAT2* levels were assessed using microarray data from ADNI. Statistical significance was determined using ANOVA (*p* < 0.05 considered significant). (C) Association of *LPCAT2* SNPs with pain experience. The association of the top three *LPCAT2* SNPs (rs9922534, rs1362399, and rs837550) with pain experience was evaluated in non‐E4 carriers. Statistical significance was assessed using a chi‐square test. (D) Association of *LPCAT2* SNPs with AD risk. Numbers in parentheses after group labels indicate sample sizes.

To investigate the role of LPCAT2 in linking pain susceptibility and AD risk, we aim to identify whether there are functional *LPCAT2* SNPs that may mediate pain susceptibility and AD risk (Figure 4A). For this end, we first investigate whether there are *LPCAT2* SNPs that associate with its gene expression, given that functional SNPs often modulate the expression of immune‐related genes (Consortium 2015; Fairfax et al. 2012; Lappalainen and MacArthur 2021; Mostafavi et al. 2023). We performed an expression quantitative trait loci (eQTL) analysis using the ADNI dataset. We identified 11 *LPCAT2* SNPs significantly associated with its expression in the peripheral blood of non‐E4 individuals (Figure 4B; Table 1, columns 3) and then assessed their association with pain susceptibility (Figure 4C; Table 1, columns 4–7). Genetic association analysis revealed a significant correlation between these SNPs and pain susceptibility in non‐E4 males, whereas no significant association was observed in females or *APOE4* males (Tables S4–S6). This finding underscores a distinct genetic predisposition to pain susceptibility in non‐E4 males during the progression to AD.

**TABLE 1:** Association of *LPCAT2* SNPs pain experience and AD risk in non‐E4 male in the ADNI database.

SNPs	A1/A2	*LPCAT2* levels	Pain experience				AD risk			
		*p*	F_U (117)	F_A (147)	CHISQ	*p*	F_U (145)	F_A (102)	CHISQ	*p*
rs9922534	C/T	< 0.001	0.534	0.405	8.781	**0.003**	0.510	0.387	7.306	**0.007**
rs837550	G/A	< 0.001	0.402	0.286	7.845	**0.005**	0.400	0.260	10.460	**0.001**
rs1362399	C/T	< 0.001	0.513	0.391	7.809	**0.005**	0.497	0.363	8.697	**0.003**
rs2216058	C/T	< 0.001	0.534	0.415	7.439	**0.006**	0.514	0.392	7.125	**0.008**
rs1558664	C/A	< 0.001	0.509	0.391	7.276	**0.007**	0.497	0.358	9.355	**0.002**
rs1898414	T/C	< 0.001	0.509	0.391	7.276	**0.007**	0.497	0.358	9.355	**0.002**
rs1558666	A/G	< 0.001	0.419	0.527	6.136	**0.013**	0.435	0.554	6.841	**0.009**
rs4402561	C/T	< 0.001	0.521	0.422	5.194	**0.023**	0.510	0.392	6.731	**0.009**
rs11647464	T/C	< 0.001	0.500	0.401	5.133	**0.023**	0.479	0.387	4.116	**0.042**
rs1393261	T/C	< 0.001	0.419	0.507	4.053	**0.044**	0.435	0.525	3.895	**0.048**
rs2192855	A/G	< 0.001	0.410	0.497	3.913	**0.048**	0.424	0.520	4.389	**0.036**

*Note: LPCAT2* levels: the association between SNPs and blood *LPCAT2* levels. Pain experience: the association between SNPs and self‐reported musculoskeletal pain perception. AD risk: the association between SNPs and AD risk. A1: A1 allele, A2: A2 allele; F_A: frequency of the effect allele in subjects with high pain perception or AD diagnosis. F_U: frequency of the effect allele in subjects with low pain perception or CN status; CHISQ: basic allelic test chi‐square (1df), *p*: *p* value for the CHISQ test, with values less than 0.05 considered statistically significant and highlighted in orange bold.

To strengthen the link between *LPCAT2* SNPs and pain experience, we extended our analysis to include datasets from different ethnic groups, specifically the Taiwan BioBank cohort (https://www.twbiobank.org.tw/). Consistent with the ADNI results, our genetic association analysis using the dataset identified a significant association between the *LPCAT2* SNP rs183112 and pain experience in non‐E4 male subjects (*p* = 0.041). Additionally, two other SNPs, rs865094 and rs243843, showed modest correlations (*p* = 0.058 and 0.089, respectively) (Table S7, columns 7 through 10). These findings, derived from analyses across two distinct ethnic cohorts, underscore the genetic connection between *LPCAT2* variants and pain susceptibility, particularly in non‐E4 individuals. Our results align with previous research demonstrating the pain‐relieving effects of *LPCAT2* knockout (Yamamoto et al. 2024), further validating LPCAT2's role in pain perception.

We then assessed whether 11 *LPCAT2* SNPs were associated with AD risk across multiple datasets. In the ADNI cohort, several of these SNPs showed significant associations with increased AD risk specifically in non‐E4 males (Figure 4D and Table 1, columns 8–11), whereas no such associations were observed in non‐E4 females or *APOE4* males (Tables S4–S6). Supporting this pattern, analysis of the Taiwan BioBank dataset revealed that SNP rs183112 was significantly associated with MMSE scores, particularly in non‐E4 males (*p* = 0.017), with rs865094 and rs243843 showing suggestive trends (Table S7). Consistent findings were also observed in the ROSMAP and MayoLOADGWAS cohorts, where several *LPCAT2* SNPs were significantly associated with AD‐related phenotypes in non‐E4 males (*p* values ranging from 0.027 to 0.041), after adjusting for age (Table S8). These findings collectively underscore a consistent and convergent pattern across three independent cohorts, indicating that *LPCAT2* genetic variation is strongly associated with AD risk and related traits in a genotype‐ and sex‐specific manner.

### 3.5 Mendelian Randomization Analysis Identifies Causal Effects of *LPCAT2* Variants on Pain Susceptibility and AD Risk

**FIGURE 5:**
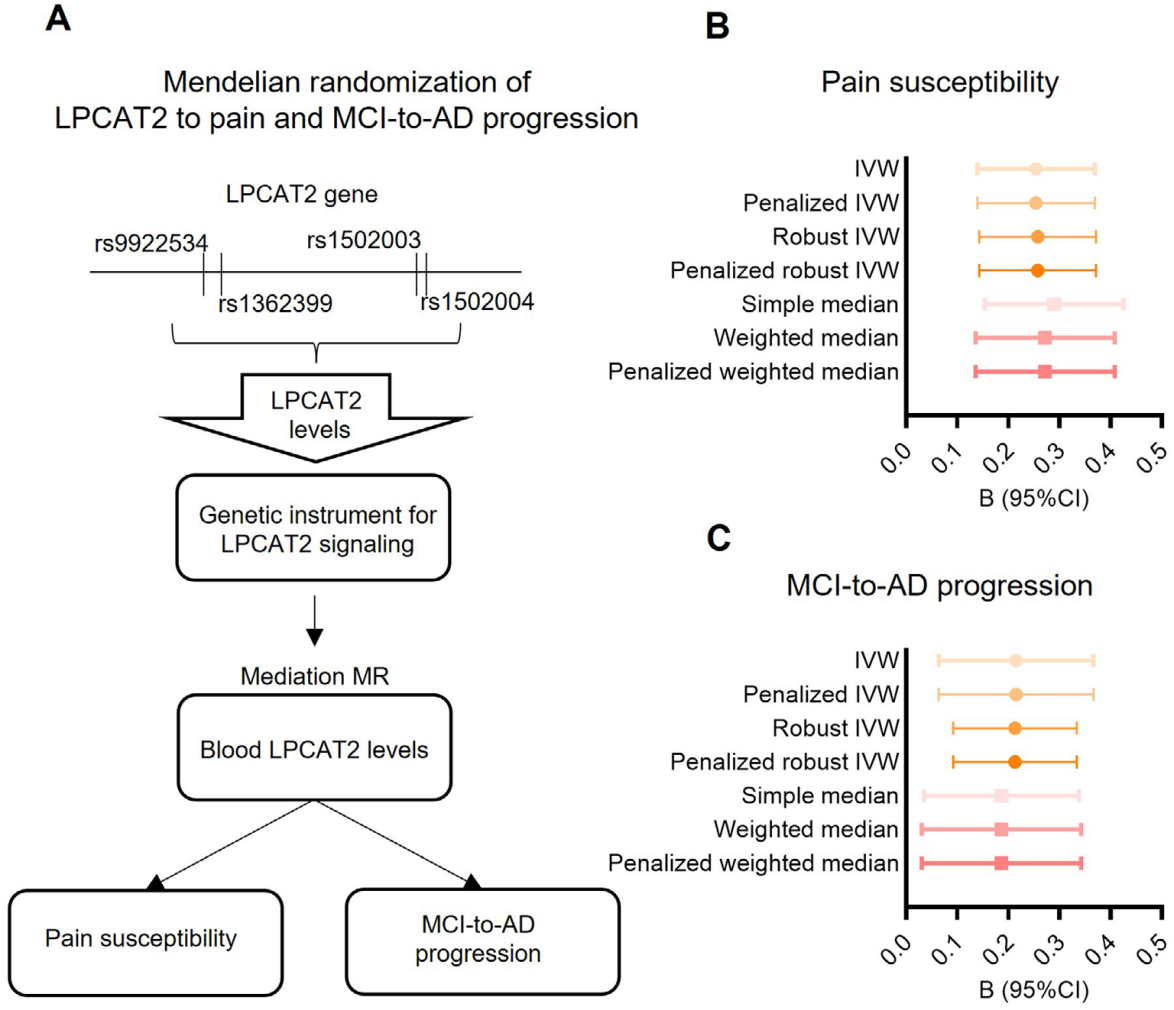
Genetic instrument‐based Mendelian randomization linking LPCAT2 signaling to pain susceptibility and MCI‐to‐AD progression. (A) Schematic overview of the two‐sample Mendelian randomization (MR) analysis design. Genetic variants associated with *LPCAT2* expression were used as instrumental variables to infer the causal effect of LPCAT2 signaling on two downstream phenotypes: pain susceptibility and progression from MCI to AD. *LPCAT2* expression levels in blood were derived from ADNI datasets in non‐E4 individuals. Sample sizes were *n* = 601 for the pain susceptibility analysis and *n* = 173 for the MCI‐to‐AD progression analysis. (B) MR estimates of the causal effect of LPCAT2 signaling on pain susceptibility using seven methods, including four IVW‐based approaches (standard IVW, penalized IVW, robust IVW, and penalized robust IVW) and three median‐based estimators (simple median, weighted median, and penalized weighted median). (C) MR estimates of the causal effect of LPCAT2 signaling on MCI‐to‐AD progression using the same set of MR approaches. Horizontal bars indicate 95% confidence intervals.

To explore potential causal relationships beyond observed correlations, we applied multiple MR approaches to assess whether genetic variants of *LPCAT2* contribute to pain susceptibility and Alzheimer's disease risk (Figure 5A). The inverse‐variance weighted (IVW) method demonstrated a significant positive association (*β* = 0.254, SE = 0.059, *p* = 1.43 × 10^−5^), indicating that a higher genetic predisposition to pain susceptibility (Figure 5B and Table S9). This association was consistently supported across multiple MR models, including penalized IVW, robust IVW, and penalized robust IVW. In addition, median‐based estimators yielded consistent and significant effects, with the weighted median, penalized weighted median, and simple median models all demonstrating statistically significant associations, further strengthening the robustness of the inferred causal relationship. These consistent findings across multiple MR approaches strengthen the causal interpretation of the observed relationship.

We further applied the same set of MR methods to evaluate the potential impact of *LPCAT2* genetic signaling on the progression from MCI to AD (Figure 5C and Table S10). The IVW model revealed a significant positive association (*β* = 0.215, SE = 0.077, *p* = 5.27 × 10^−3^), suggesting that genetically determined LPCAT2 activity may increase the risk of MCI‐to‐AD conversion. This result remained robust and directionally consistent across penalized and robust IVW methods (*β* ≈ 0.213, *p* = 5.46 × 10^−4^). Supporting evidence was also observed in the median‐based models, though with slightly attenuated effect sizes. Together, these findings provide converging genetic evidence from multiple MR approaches linking pain susceptibility and LPCAT2 signaling to increased risk of AD progression.

## 4 Discussion

Although *APOE4* is a well‐established risk factor that contributes to disease stratification and prevention, it accounts for only 40%–65% of AD cases (Farrer et al. 1997; Serrano‐Pozo et al. 2021). Consequently, identifying genetic risk factors in non‐E4 individuals is crucial for fully elucidating AD's etiology. In this study, we reveal a significant causal link between pain susceptibility and AD onset, particularly in non‐E4 males. Our findings provide clinical and genetic evidence that LPCAT2 is a key risk and prognostic factor for MCI‐to‐AD progression in this subgroup. Moreover, we uncover a genotype‐ and sex‐specific relationship between *LPCAT2* expression, cognitive decline, and pain susceptibility, underscoring the importance of considering both *APOE* genetic background and sex in AD pathophysiology. This insight highlights LPCAT2's potential as a novel therapeutic target and underscores the importance of addressing genetic variations to enable prognosis and personalized treatment strategies, ultimately broadening the scope of care for diverse populations affected by AD.

Based on our findings that *LPCAT2* expression levels in peripheral blood are key predictors of pain susceptibility and AD onset, we identified several functional *LPCAT2* SNPs that may regulate its expression. Importantly, these SNPs are associated with both pain susceptibility and an increased risk of AD, particularly in non‐E4 males. We also validated these findings in an independent ethnic cohort, which confirmed a significant association between *LPCAT2* SNPs, pain susceptibility, and cognitive decline in the same subpopulation, enhancing our understanding of AD in underrepresented populations. These results suggest a genetic predisposition to pain susceptibility in non‐E4 individuals during AD progression. Furthermore, they highlight *LPCAT2* SNPs and blood transcript levels as promising prognostic biomarkers for predicting AD onset in non‐E4 males during the MCI stage. To establish causality, we performed MR analysis, which revealed a direct link between *LPCAT2* variants, pain susceptibility, and AD risk in non‐E4 males. This further supports the causal role of *LPCAT2* variants in both AD pathogenesis and pain modulation.

Given AD's neurodegenerative nature, we examined LPCAT2 protein levels in brain tissues. Analysis of postmortem samples showed significantly elevated LPCAT2 protein levels in the hippocampus of non‐E4 male AD patients. Consistently, APP/PS1 AD mice exhibited a marked increase in hippocampal LPCAT2 expression, while no significant changes were observed in the cortex. Notably, LPCAT2 is predominantly expressed in the CNS microglial cells (Okubo et al. 2012; Yamamoto et al. 2024), which may explain the regional specificity of its upregulation, as the hippocampus is densely populated with microglia and therefore more susceptible to changes in LPCAT2 levels (Choi and Won 2011; Lawson et al. 1990).

LPCAT2 is a key enzyme that reacylates LPC into phosphatidylcholine (PC), thereby remodeling membrane lipid composition. Beyond lipid metabolism, LPCAT2 regulates pain sensitivity and inflammation, particularly through the TLR4‐activated interferon (IFN) cascade (Abate et al. 2020; Huang et al. 1999; Poloamina et al. 2024). In the CNS, TLR4 activation induces IFN signaling that shifts microglia from resting to activated states (Papageorgiou et al. 2016). This transition enhances phagocytosis and defense acutely, but its persistence drives chronic neuroinflammation and neural impairment. LPCAT2's immunomodulatory effects may be influenced by sex and *APOE* genotype through three interconnected axes: LPC substrate availability, microglial responsiveness, and hormonal regulation of the TLR4–IFN pathway. Substrate supply is shaped by sex‐dependent hepatic phospholipid metabolism (Graham et al. 1991) and APOE‐driven changes in circulating LPC (Law et al. 2023), thereby affecting PC remodeling and downstream signaling. Microglia, both targets and mediators of LPCAT2 (Scholz and Eder 2017; Yamamoto et al. 2024), differ in number, gene expression, and activation thresholds by sex (Han et al. 2021), whereas APOE defines baseline immune state (Ferrari‐Souza et al. 2023). Thus, LPCAT2 activity may yield distinct neuroimmune phenotypes across sex‐genotype contexts. Hormones provide an additional layer: estrogen enhances IFN gene activity (Fox et al. 1991; Karpuzoglu‐Sahin et al. 2001), can suppress TLR4 activation (Meng et al. 2023), and modulates phospholipid metabolism, altering LPC/PC ratios. These actions suggest females may sustain higher basal IFN levels, potentially dampening LPCAT2‐driven IFN amplification during the transition from normal cognition to MCI. In summary, sex and APOE genotype shape LPCAT2‐related immune signaling in MCI by modulating substrate availability, cellular sensitivity, and hormonal regulation of lipid–immune interactions, contributing to sex‐ and genotype‐dependent differences in pain sensitivity and neurodegenerative progression.

Although our findings are promising, several limitations should be considered. First, although MR suggests causality, validation in animal models is needed for experimental confirmation. Second, our study focused on non‐E4 males, limiting generalizability to other genetic backgrounds and females. Notably, females exhibit higher *LPCAT2* expression and increased pain levels, which may mask detectable differences and reduce LPCAT2's impact on AD progression. Third, integrating data from multiple sources introduces variability, highlighting the need for standardized data collection to improve robustness. Fourth, the use of MMSE scores from the Taiwan Biobank, while correlated with MCI/AD, does not definitively indicate these conditions. Lastly, the mechanisms underlying sex‐ and *APOE* genotype‐related differences remain unclear, requiring further investigation into LPCAT2's role in AD across diverse populations.

Our findings establish LPCAT2 as a pivotal risk factor for pain perception and AD onset in non‐E4 males, emphasizing the critical need for personalized approaches in AD research and therapy. By uncovering genotype‐ and sex‐specific associations, this study highlights LPCAT2 as a promising biomarker for predicting disease progression. Furthermore, the potential of targeting the LPCAT2 pathway offers new avenues for developing tailored treatment strategies, particularly for non‐E4 males—an understudied subgroup in the AD field. Future research should investigate the interplay between *LPCAT2* genetic variants, expression levels, and inflammation, as well as their interactions with other genetic and environmental factors, to provide deeper insights into their role in cognitive decline. By linking lipid metabolism enzyme, pain modulation, and AD pathogenesis, this study advances the field toward precision medicine and underscores the importance of addressing subgroup‐specific vulnerabilities in AD progression and treatment.

## Author Contributions

R.H.L. and J.L.J. conceptualized and supervised the study. R.H.C., Y.C.C., and K.H.L. performed genetic association and statistical analyses. Y.C.C. and C.N.L. conducted bioinformatics and multi‐omics integration. W.Y.P. and R.H.L. carried out animal experiments and tissue analyses. All authors contributed to data interpretation, critically reviewed the manuscript, and approved the final version for submission.

## Conflicts of Interest

The authors declare no conflicts of interest.

## Supporting information


**Figure S1:** Association of *LPCAT2* and other blood transcript levels with pain experience in non‐E4 males.
**Figure S2:** Association of candidate transcript levels with the progression from MCI to AD in non‐E4 carriers.
**Figure S3:** Western blot analysis of LPCAT2 levels in hippocampal tissues from postmortem AD samples.
**Table S1:** Baseline characteristics of control (CN) and MCI (MCI) in ADNI study.
**Table S2:** Baseline characteristics of subjects in Taiwan BioBank.
**Table S3:** Blood microarray analysis in non‐E4 MCI males.
**Table S4:** Association of *LPCAT2* SNPs with pain experience and AD risk in *APOE4* male in the ADNI database.
**Table S5:** Association of *LPCAT2* SNPs with pain experience and AD risk in non‐E4 female in the ADNI database.
**Table S6:** Association of *LPCAT2* SNPs with pain experience and AD risk in *APOE4* female in the ADNI database.
**Table S7:** Association of *LPCAT2* SNPs with pain experience and MMSE in male subjects with non‐E4 genotype in Taiwan Biobank.
**Table S8:** Association of *LPCAT2* SNPs with pain experience and AD risk in the ROSMAP and MayoLOADGWAS dataset.
**Table S9:** Mendelian randomization analysis of *LPCAT2* and pain susceptibility.
**Table S10:** Mendelian randomization analysis of *LPCAT2* and MCI‐to‐AD progression.

## Data Availability

Human data used in this study were obtained from the Alzheimer's Disease Neuroimaging Initiative, the Religious Orders Study and Memory and Aging Project, and the Taiwan Biobank, all under approved data use agreements. Access to these datasets requires application and approval from the respective data repositories. Animal experimental data and additional study materials generated during this study are available from the corresponding authors upon reasonable request.
