# Supplementary material for: Sex‐ and APOE Genotype–Dependent Pain Susceptibility and Alzheimer's Risk Mediated by the Lipid Metabolism Enzyme LPCAT2
Source: Aging Cell. 2025 Sep 17;24(11):e70234. doi: 10.1111/acel.70234 (PMC12611278; doi:10.1111/acel.70234)
Supplement: Supplementary file 1 — Figure S1: Association of LPCAT2 and other blood transcript levels with pain experience in non‐E4 males. Figure S2: Association of candidate transcript levels with the progression from MCI to AD in non‐E4 carriers. Figure S3: Western blot analysis of LPCAT2 levels in hippocampal tissues from postmortem AD samples. Table S1: Baseline characteristics of control (CN) and MCI (MCI) in ADNI study. Table S2: Baseline characteristics of subjects in Taiwan BioBank. Table S3: Blood microarray analysis in non‐E4 MCI males. Table S4: Association of LPCAT2 SNPs with pain experience and AD risk in APOE4 male in the ADNI database. Table S5: Association of LPCAT2 SNPs with pain experience and AD risk in non‐E4 female in the ADNI database. Table S6: Association of LPCAT2 SNPs with pain experience and AD risk in APOE4 female in the ADNI database. Table S7: Association of LPCAT2 SNPs with pain experience and MMSE in male subjects with non‐E4 genotype in Taiwan Biobank. Table S8: Association of LPCAT2 SNPs with pain experience and AD risk in the ROSMAP and MayoLOADGWAS dataset. Table S9: Mendelian randomization analysis of LPCAT2 and pain susceptibility. Table S10: Mendelian randomization analysis of LPCAT2 and MCI‐to‐AD progression. [file ACEL-24-e70234-s001.docx]

**Supplementary Information**

**The supplementary material includes:**

Fig. S1. Association of *LPCAT2* and other blood transcript levels with pain susceptibility in nonE4 males.

Fig. S2. Association of candidate transcript levels with the progression from MCI to AD in nonE4 carriers.

Fig. S3. Western blot analysis of LPCAT2 levels in hippocampal tissues from post-mortem AD samples.

Table S1. Baseline characteristics of control (CN) and MCI (MCI) in ADNI study.

Table S2. Baseline characteristics of subjects in Taiwan BioBank.

Table S3. Blood microarray analysis in nonE4 MCI males.

Table S4. Association of *LPCAT2* SNPs with pain experience and AD risk in *APOE4* male in the ADNI database.

Table S5. Association of *LPCAT2* SNPs with pain experience and AD risk in nonE4 female in the ADNI database.

Table S6. Association of *LPCAT2* SNPs with pain experience and AD risk in *APOE4* female in the ADNI database.

Table S7. Association of *LPCAT2* SNPs with pain experience and MMSE in male subjects with nonE4 genotype in Taiwan Biobank.

Table S8 Association of LPCAT2 SNPs with pain experience and AD risk in the ROSMAP database.

Table S9. Mendelian randomization analysis of *LPCAT2* and pain perception.

Table S10. Mendelian randomization analysis of *LPCAT2* and MCI-to-AD progression.

**
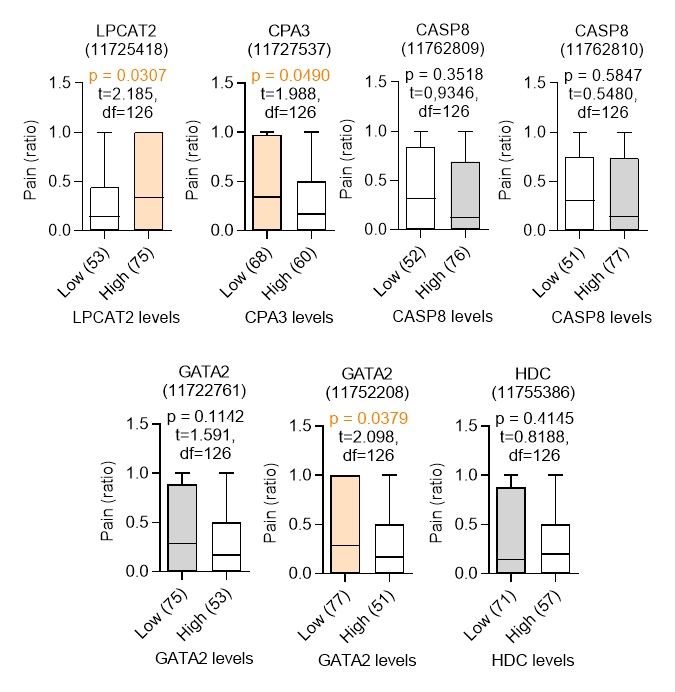
Figure S1**

**Fig. S1. Association of *LPCAT2* and other blood transcript levels with pain experience in nonE4 males.** Blood transcript levels of *LPCAT2* and additional candidate genes were analyzed for their association with pain experience in nonE4 males. Transcript levels were categorized as low or high based on the mean expression levels in individuals with pain experience. Statistical significance was determined using an unpaired t-test, with p < 0.05 considered significant. Numbers in parentheses after group labels indicate sample sizes.


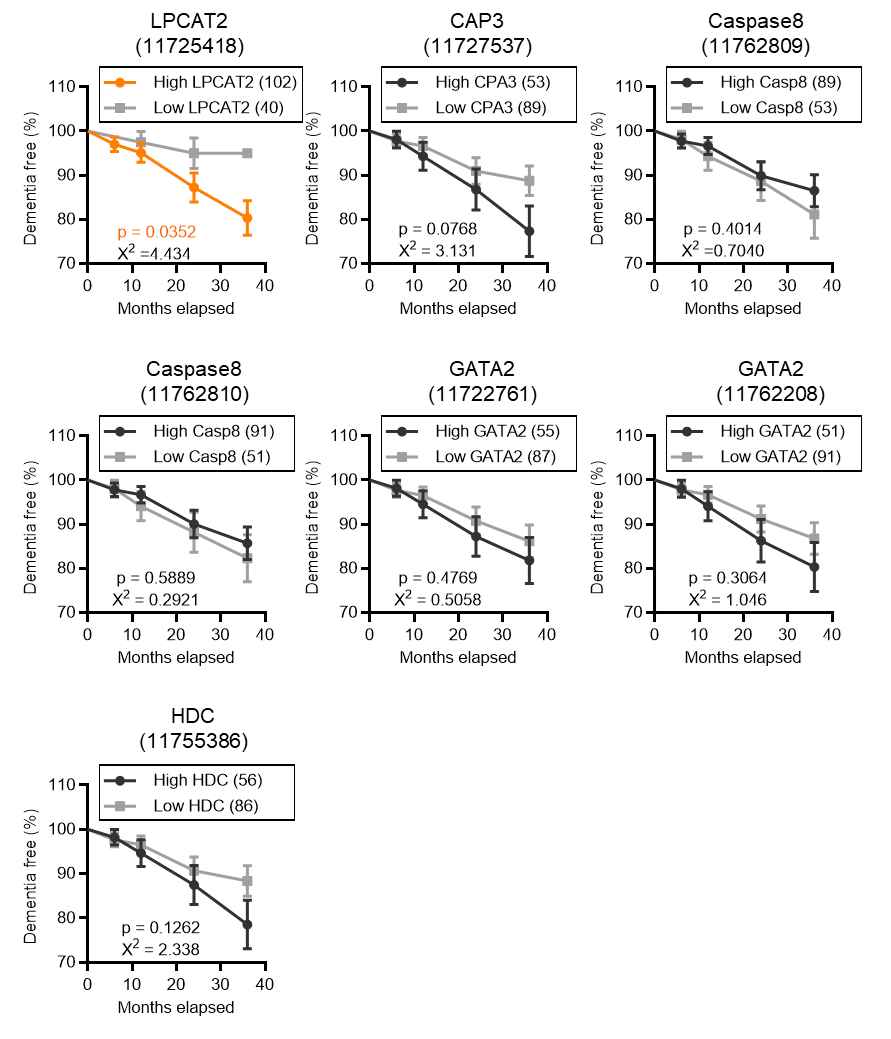
**Figure S2**

**Fig. S2. Association of candidate transcript levels with the progression from MCI to AD in nonE4 carriers.** The relationship between candidate transcript levels and the transition speed from MCI to AD was analyzed in nonE4 carriers. Transcript levels were categorized as low or high based on the mean expression levels in individuals with pain experience. Kaplan-Meier analysis was performed using the two-sided log-rank test to assess differences in progression rates. Statistical significance was defined as p < 0.05. Numbers in parentheses after group labels indicate sample sizes.


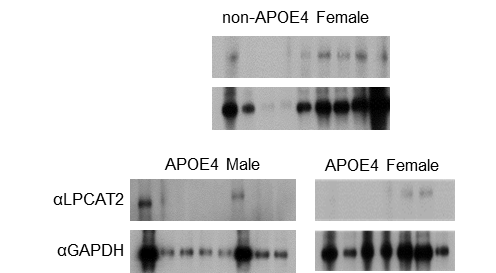
Figure S3

**Fig. S3. Western blot analysis of LPCAT2 levels in hippocampal tissues from post-mortem AD samples.** LPCAT2 protein levels were detected using specific antibodies, with GAPDH as a loading control.

**Table S1.** **Baseline characteristics of control (CN) and MCI (MCI) in ADNI study.**

|  | NonE4 Male | | NonE4 Female | |
| --- | --- | --- | --- | --- |
|  | CN | MCI | CN | MCI |
| n | 180 | 196 | 191 | 100 |
| Age | 74.87 ± 5.75 | 75.13 ± 8.03 | 73.24 ± 6.34 | 74.46 ± 8.45 |
| ABETA | 1288.01 ± 412.84 | 998.61 ± 423.31 | 1263.52 ± 431.32 | 1114.27 ± 442.32 |
| TAU | 225.34 ± 78.55 | 264.23 ± 116.14 | 234.41 ± 96.5 | 275.59 ± 136.96 |
| PTAU | 20.56 ± 8.12 | 25.21 ± 12.73 | 21.11 ± 9.13 | 26.42 ± 15.17 |
| CDRSB | 0.02 ± 0.1 | 1.55 ± 0.91 | 0.04 ± 0.13 | 1.55 ± 0.9 |
| ADAS13 | 10.11 ± 4.34 | 17.56 ± 5.88 | 7.81 ± 3.89 | 16.2 ± 7.59 |
|  | *APOE4* Male |  | *APOE4* Female |  |
|  | CN | MCI | CN | MCI |
| n | 64 | 198 | 78 | 146 |
| Age | 73.79 ± 6.89 | 74.12 ± 6.6 | 72.38 ± 5.16 | 71.3 ± 6.87 |
| ABETA | 905.34 ± 441.17 | 673.53 ± 325.18 | 991.66 ± 458.75 | 746.26 ± 295.03 |
| TAU | 255.87 ± 89.35 | 317.42 ± 122.06 | 256.47 ± 98.74 | 385.78 ± 156.86 |
| PTAU | 24.2 ± 9.25 | 32.06 ± 14.09 | 24.88 ± 11.17 | 38.51 ± 16.8 |
| CDRSB | 0.05 ± 0.17 | 1.73 ± 1 | 0.02 ± 0.1 | 1.75 ± 0.83 |
| ADAS13 | 10.25 ± 4.81 | 19.62 ± 6.25 | 8.38 ± 4.31 | 19.85 ± 6.34 |

NonE4: Participants who do not carry the *APOE4* allele; *APOE4*: Participants who carry at least one copy of the *APOE4* allele; CN: Control subjects; MCI: Mild Cognitive Impairment; n: Sample size of each subgroup; Age: Mean age ± standard deviation (years); ABETA: Cerebrospinal fluid (CSF) Aβ42 levels (pg/mL), mean ± standard deviation. TAU: CSF total Tau levels (pg/mL), mean ± standard deviation; PTAU: CSF phosphorylated Tau-181 levels (pg/mL), mean ± standard deviation; CDRSB: Clinical Dementia Rating Sum of Boxes (CDRSB) score, mean ± standard deviation. ADAS13: Alzheimer’s Disease Assessment Scale-Cognitive Subscale (13-item version), mean ± standard deviation.

**Table S2. Baseline characteristics of subjects in Taiwan BioBank**.

| Characteristic | nonE4 male | nonE4 Female | E4 male | E4 Female |
| --- | --- | --- | --- | --- |
| Mean ± SD (n) | (626) | (1327) | (141) | (280) |
| Age | 63.92 ± 2.792 | 63.3 ± 2.651 | 63.91 ± 2.842 | 63.23 ± 2.602 |
| Education | 13.30 ± 3.644 | 11.43 ± 4.034 | 13.71 ± 3.335 | 11.78 ± 3.960 |
| Articular pain | 153 (24.4%) | 449 (33.8%) | 35 (24.8%) | 99 (35.4%) |
| MMSE | 27.43 ± 2.123 | 27.01 ± 2.489 | 27.55 ± 2.209 | 27.33 ± 2.203 |

NonE4: Participants who do not carry the *APOE4* allele; *APOE4*: Participants who carry at least one copy of the *APOE4* allele; n: Sample size of each subgroup; Age: Years of age; Education: Years of education; Articular pain: Number and percentage of individuals experiencing joint pain; MMSE: Mini-Mental State Examination score. Numbers in parentheses after group labels indicate sample sizes.

**Table S3. Blood microarray analysis in nonE4 MCI males.**

| ProbeSet | CN (n = 90) | MCI (n = 133) | p | t | perm_p |
| --- | --- | --- | --- | --- | --- |
| CASP8_11762810_x_at | 4.394 ± 1.646 | 5.115 ± 1.563 | **0.001** | -3.291 | **0.001** |
| CASP8_11762809_at | 4.086 ± 1.721 | 4.787 ± 1.696 | **0.003** | -2.995 | **0.004** |
| LPCAT2_11725418_a_at | 5.820 ± 1.211 | 6.280 ± 1.107 | **0.004** | -2.918 | **0.005** |
| GATA2_11722761_a_at | 6.284 ± 1.141 | 5.85 ± 1.123 | **0.006** | 2.797 | **0.006** |
| HDC_11755386_a_at | 4.999 ± 1.202 | 4.598 ± 1.100 | **0.011** | 2.564 | **0.011** |
| CPA3_11727537_at | 4.724 ± 1.409 | 4.300 ± 1.314 | **0.023** | 2.284 | **0.023** |
| GATA2_11752208_a_at | 4.967 ± 1.393 | 4.548 ± 1.300 | **0.024** | 2.279 | **0.023** |

CN, Cognitively Normal; MCI, Mild Cognitive Impairment; p, p-value; t, t-value; perm_p: Permutation test-derived p-value. P-values less than 0.05 are considered statistically significant and are highlighted in orange bold. Numbers in parentheses after group labels indicate sample sizes.

**Table S4. Association of *LPCAT2* SNPs with pain experience and AD risk in *APOE4* male in the ADNI database.**

|  |  | Pain experience | | | | AD risk | | | |
| --- | --- | --- | --- | --- | --- | --- | --- | --- | --- |
| SNP | A1/A2 | F_U  (89) | F_A  (95) | CHISQ | p | F_U  (125) | F_A  (34) | CHISQ | p |
| rs9922534 | C / T | 0.511 | 0.490 | 0.174 | 0.677 | 0.484 | 0.559 | 1.197 | 0.274 |
| rs837550 | G / A | 0.343 | 0.358 | 0.093 | 0.760 | 0.356 | 0.338 | 0.074 | 0.786 |
| rs1362399 | C / T | 0.483 | 0.479 | 0.006 | 0.936 | 0.476 | 0.500 | 0.123 | 0.725 |
| rs2216058 | C / T | 0.455 | 0.453 | 0.002 | 0.963 | 0.460 | 0.485 | 0.138 | 0.711 |
| rs1558664 | C / A | 0.483 | 0.463 | 0.147 | 0.701 | 0.472 | 0.485 | 0.038 | 0.846 |
| rs1898414 | T / C | 0.483 | 0.463 | 0.147 | 0.701 | 0.472 | 0.485 | 0.038 | 0.846 |
| rs1558666 | A / G | 0.427 | 0.484 | 1.214 | 0.271 | 0.468 | 0.412 | 0.682 | 0.409 |
| rs4402561 | C / T | 0.449 | 0.474 | 0.217 | 0.641 | 0.460 | 0.515 | 0.642 | 0.423 |
| rs11647464 | T / C | 0.427 | 0.458 | 0.356 | 0.551 | 0.440 | 0.500 | 0.777 | 0.378 |
| rs1393261 | T / C | 0.472 | 0.484 | 0.056 | 0.813 | 0.484 | 0.427 | 0.710 | 0.399 |
| rs2192855 | A / G | 0.449 | 0.505 | 1.148 | 0.284 | 0.484 | 0.412 | 1.120 | 0.290 |

Pain experience: The association between SNPs and self-reported musculoskeletal pain perception. AD risk: The association between SNPs and AD risk. A1: A1 allele, A2: A2 allele; F_A: Frequency of the effect allele in subjects with high pain perception or AD diagnosis. F_U: Frequency of the effect allele in subjects with low pain susceptibility or CN status; CHISQ: basic allelic test chi-square (1df), p: p-value for CHISQ. Numbers in parentheses after group labels indicate sample sizes.

**Table S5. Association of *LPCAT2* SNPs with pain experience and AD risk in nonE4 female in the ADNI database.**

|  |  | Pain experience | | | | AD risk | | | |
| --- | --- | --- | --- | --- | --- | --- | --- | --- | --- |
| SNP | A1/A2 | F_U  (109) | F_A  (107) | CHISQ | p | F_U  (103) | F_A  (99) | CHISQ | p |
| rs9922534 | C / T | 0.491 | 0.421 | 2.149 | 0.143 | 0.4417 | 0.4646 | 0.2136 | 0.6439 |
| rs837550 | G / A | 0.367 | 0.304 | 1.936 | 0.164 | 0.3107 | 0.3485 | 0.6534 | 0.4189 |
| rs1362399 | C / T | 0.477 | 0.402 | 2.478 | 0.115 | 0.4223 | 0.4444 | 0.2011 | 0.6538 |
| rs2216058 | C / T | 0.440 | 0.383 | 1.458 | 0.227 | 0.3981 | 0.4242 | 0.286 | 0.5928 |
| rs1558664 | C / A | 0.463 | 0.393 | 2.210 | 0.137 | 0.4029 | 0.4444 | 0.7134 | 0.3983 |
| rs1898414 | T / C | 0.463 | 0.393 | 2.210 | 0.137 | 0.4029 | 0.4444 | 0.7134 | 0.3983 |
| rs1558666 | A / G | 0.459 | 0.523 | 1.806 | 0.179 | 0.5097 | 0.4798 | 0.3613 | 0.5478 |
| rs4402561 | C / T | 0.477 | 0.411 | 1.896 | 0.169 | 0.4175 | 0.4596 | 0.7276 | 0.3937 |
| rs11647464 | T / C | 0.445 | 0.397 | 1.010 | 0.315 | 0.3981 | 0.4444 | 0.8913 | 0.3451 |
| rs1393261 | T / C | 0.482 | 0.467 | 0.089 | 0.765 | 0.4563 | 0.4848 | 0.3301 | 0.5656 |
| rs2192855 | A / G | 0.459 | 0.495 | 0.576 | 0.448 | 0.4951 | 0.4694 | 0.2669 | 0.6054 |

Pain experience: The association between SNPs and self-reported musculoskeletal pain perception. AD risk: The association between SNPs and AD risk. A1: A1 allele, A2: A2 allele; F_A: Frequency of the effect allele in subjects with high pain perception or AD diagnosis. F_U: Frequency of the effect allele in subjects with low pain susceptibility or CN status; CHISQ: basic allelic test chi-square (1df), p: p-value for CHISQ. Numbers in parentheses after group labels indicate sample sizes.

**Table S6. Association of *LPCAT2* SNPs with pain experience and AD risk in *APOE4* female in the ADNI database.**

|  |  | Pain experience | | | | AD risk | | | |
| --- | --- | --- | --- | --- | --- | --- | --- | --- | --- |
| SNP | A1/A2 | F_U  (79) | F_A  (69) | CHISQ | p | F_U  (88) | F_A  (41) | CHISQ | p |
| rs9922534 | C / T | 0.424 | 0.442 | 0.097 | 0.755 | 0.415 | 0.415 | 0.000 | 0.998 |
| rs837550 | G / A | 0.361 | 0.333 | 0.244 | 0.621 | 0.352 | 0.317 | 0.308 | 0.579 |
| rs1362399 | C / T | 0.437 | 0.406 | 0.289 | 0.591 | 0.415 | 0.402 | 0.035 | 0.851 |
| rs2216058 | C / T | 0.456 | 0.377 | 1.883 | 0.170 | 0.421 | 0.402 | 0.075 | 0.785 |
| rs1558664 | C / A | 0.430 | 0.391 | 0.464 | 0.496 | 0.409 | 0.390 | 0.083 | 0.774 |
| rs1898414 | T / C | 0.430 | 0.391 | 0.464 | 0.496 | 0.409 | 0.390 | 0.083 | 0.774 |
| rs1558666 | A / G | 0.462 | 0.522 | 1.051 | 0.305 | 0.494 | 0.500 | 0.007 | 0.932 |
| rs4402561 | C / T | 0.449 | 0.399 | 0.778 | 0.378 | 0.421 | 0.402 | 0.075 | 0.785 |
| rs11647464 | T / C | 0.462 | 0.377 | 2.193 | 0.139 | 0.426 | 0.390 | 0.297 | 0.586 |
| rs1393261 | T / C | 0.430 | 0.536 | 3.307 | 0.069 | 0.483 | 0.500 | 0.065 | 0.799 |
| rs2192855 | A / G | 0.418 | 0.515 | 2.775 | 0.096 | 0.460 | 0.488 | 0.171 | 0.679 |

Pain experience: The association between SNPs and self-reported musculoskeletal pain perception. AD risk: The association between SNPs and AD risk. A1: A1 allele, A2: A2 allele; F_A: Frequency of the effect allele in subjects with high pain perception or AD diagnosis. F_U: Frequency of the effect allele in subjects with low pain susceptibility or CN status; CHISQ: basic allelic test chi-square (1df), p: p-value for CHISQ. Numbers in parentheses after group labels indicate sample sizes.

**Table S7. Association of *LPCAT2* SNPs with pain experience and MMSE in male subjects with nonE4 genotype in Taiwan Biobank.**

| SNP | A1/A2 | MAF | LPCAT2 levels | | | Pain (n = 1828) | | | | MMSE (n = 741) | | | | |
| --- | --- | --- | --- | --- | --- | --- | --- | --- | --- | --- | --- | --- | --- | --- |
|  |  |  | Higher level allele | p | NES | Risk allele | F_U | F_A | p | Risk allele | A1A1 | A1A2 | A2A2 | p |
|  |  |  |  |  |  |  | (513) | (1315) |  |  |  |  |  |  |
| rs865094 | G/A | 0.335 | A | 3.20E-05 | 0.19 | A | 0.311 | 0.344 | 0.058 | A | 27.56 ± 2.424 | 27.35 ± 2.346 | 27.18 ± 2.636 | 0.432 |
| rs243843 | G/A | 0.426 | A | 1.60E-05 | 0.19 | A | 0.404 | 0.435 | 0.089 | A | 27.49 ± 2.172 | 27.35 ± 2.399 | 27.09 ± 2.764 | 0.264 |
| rs183112 | A/G | 0.232 | G | 4.60E-06 | 0.21 | G | 0.209 | 0.241 | **0.041** | G | 27.81 ± 2.132 | 27.58 ± 2.037 | 27.08 ± 2.721 | **0.017** |

*LPCAT2* levels: The association between SNPs and *LPCAT2* expression levels, based on the eQTL database.

Pain experience: The association between SNPs and self-reported musculoskeletal pain perception.

MMSE: The association between SNPs and cognitive function, assessed using MMSE scores. A1: A1 allele, A2: A2 allele; F_A: Frequency of the effect allele in subjects with high pain perception or AD diagnosis. F_U: Frequency of the effect allele in subjects with low pain susceptibility or CN status; CHISQ: basic allelic test chi-square (1df), p: p-value for CHISQ and p < 0.05 in orange bold indicates statistical significance. Numbers in parentheses after group labels indicate sample sizes.

**Table S8. Association of *LPCAT2* SNPs with pain experience and AD risk in the ROSMAP and MayoLOADGWAS dataset.**

| ROSMAP | | | | | | | |
| --- | --- | --- | --- | --- | --- | --- | --- |
|  |  | nonE4-male | | | nonE4-female | | |
| **SNP** | **A1** | **NMISS** | **STAT** | **P** | **NMISS** | **STAT** | **P** |
| rs4402561 | G | 278 | 7.197 | **0.027** | 663 | 1.326 | 0.515 |
| rs13337274 | A | 276 | 6.879 | **0.032** | 664 | 0.722 | 0.697 |
| rs1583587 | G | 277 | 6.632 | **0.036** | 666 | 0.695 | 0.707 |
| rs16955475 | T | 275 | 6.397 | **0.041** | 659 | 1.492 | 0.474 |
|  |  | E4-male | | | E4-female | | |
| **SNP** | **A1** | **NMISS** | **STAT** | **P** | **NMISS** | **STAT** | **P** |
| rs4402561 | G | 106 | 0.512 | 0.774 | 221 | 0.239 | 0.888 |
| rs13337274 | A | 106 | 0.923 | 0.630 | 220 | 0.582 | 0.748 |
| rs1583587 | G | 106 | 0.923 | 0.630 | 220 | 0.395 | 0.821 |
| rs16955475 | T | 104 | 2.032 | 0.362 | 218 | 0.838 | 0.658 |
| MayoLOADGWAS | | | | | | | |
|  |  | nonE4-male | | | nonE4-female | | |
| **SNP** | **A1** | **NMISS** | **STAT** | **P** | **NMISS** | **STAT** | **P** |
| rs1558666 | A | 476 | -1.70 | 0.088 | 486 | 0.036 | 0.971 |
| rs883180 | G | 483 | -2.14 | **0.032** | 495 | -0.134 | 0.893 |
| rs1502007 | A | 468 | -1.93 | 0.054 | 486 | -0.325 | 0.745 |
|  |  | E4-male | | | E4-female | | |
| **SNP** | **A1** | **NMISS** | **STAT** | **P** | **NMISS** | **STAT** | **P** |
| rs1558666 | A | 232 | 0.435 | 0.664 | 244 | 1.071 | 0.284 |
| rs883180 | G | 242 | -1.498 | 0.134 | 250 | 1.832 | 0.067 |
| rs1502007 | A | 239 | 0.889 | 0.374 | 243 | 1.850 | 0.064 |

#A1: A1 allele; NMISS: number of non-missing genotypes; STAT: coefficient t-statistic; P: p-value for the t-statistic, adjusted for age. p-values less than 0.05 are highlighted in orange bold to indicate statistical significance. Numbers in parentheses after group labels indicate sample sizes.

**Table S9. Mendelian randomization analysis of *LPCAT2* and pain susceptibility.**

| **Mendelian Randomization (*LPCAT2* vs. pain perception)** | | | | | |
| --- | --- | --- | --- | --- | --- |
| **Method** | **Estimate** | **Std Error** | **95% CI** | | **P-value** |
| Simple median | 0.28911 | 0.06913 | 0.15362 | 0.42460 | **2.89E-05** |
| Weighted median | 0.27189 | 0.06942 | 0.13583 | 0.40796 | **8.98E-05** |
| Penalized weighted median | 0.27189 | 0.06942 | 0.13583 | 0.40796 | **8.98E-05** |
| IVW | 0.25413 | 0.05857 | 0.13934 | 0.36892 | **1.43E-05** |
| Penalized IVW | 0.25413 | 0.05857 | 0.13934 | 0.36892 | **1.43E-05** |
| Robust IVW | 0.25729 | 0.05818 | 0.14326 | 0.37132 | **9.77E-06** |
| Penalized robust IVW | 0.25729 | 0.05818 | 0.14326 | 0.37132 | **9.77E-06** |

This table presents the results of Mendelian randomization (MR) analysis assessing the causal relationship between *LPCAT2* expression and pain susceptibility. Estimate: The estimated causal effect of *LPCAT2* expression on pain susceptibility. Std Error: The standard error of the causal estimate. 95% CI: The 95% confidence interval for the estimate. P-value: Indicates the statistical significance of the causal estimate; values less than 0.05 are considered significant and are highlighted in orange bold.

**Table S10. Mendelian randomization analysis of *LPCAT2* and MCI-to-AD progression.**

| **Mendelian Randomization (*LPCAT2* vs. MCI-to-AD progression)** | | | | | |
| --- | --- | --- | --- | --- | --- |
| **Method** | **Estimate** | **Std Error** | **95% CI** | | **P-value** |
| Simple median | 0.18663 | 0.07735 | 0.03502 | 0.33824 | **1.58E-02** |
| Weighted median | 0.18635 | 0.07944 | 0.03065 | 0.34204 | **1.90E-02** |
| Penalized weighted median | 0.18635 | 0.07944 | 0.03065 | 0.34204 | **1.90E-02** |
| IVW | 0.21509 | 0.07708 | 0.06401 | 0.36616 | **5.27E-03** |
| Penalized IVW | 0.21509 | 0.07708 | 0.06401 | 0.36616 | **5.27E-03** |
| Robust IVW | 0.21300 | 0.06161 | 0.09225 | 0.33375 | **5.46E-04** |
| Penalized robust IVW | 0.21300 | 0.06161 | 0.09225 | 0.33375 | **5.46E-04** |

This table presents the results of Mendelian randomization (MR) analysis assessing the causal relationship between *LPCAT2* expression and the progression from MCI to AD. Estimate: The estimated causal effect of *LPCAT2* expression on pain perception. Std Error: The standard error of the causal estimate. 95% CI: The 95% confidence interval for the estimate. P-value: Indicates the statistical significance of the causal estimate; values less than 0.05 are considered significant and are highlighted in orange bold.
